# Socioeconomic Deprivation Status and Air Pollution by PM_10_ and NO_2_: An Assessment at Municipal Level of 11 Years in Italy

**DOI:** 10.1155/2019/2058467

**Published:** 2019-01-03

**Authors:** Alice Mannocci, Immacolata Ciarlo, Valeria D'Egidio, Angela Del Cimmuto, Maria de Giusti, Paolo Villari, Giuseppe La Torre

**Affiliations:** Department of Public Health and Infectious Diseases, Sapienza University of Rome, Rome, Italy

## Abstract

The aim of this observational study was to assess the relationship between environmental risk factors and some aspects of social economic status (SES) of the population in different Italian municipalities. Nitrogen dioxide (NO_2_) and particulate matter (PM_10_) annual means were extracted from ISPRA-BRACE (environmental information system of 483 Italian municipalities, 6% of the total amount of administrative units) from 2002 to 2012. As an indicator of sociodemographic and SES data, we collected the following: resident population, foreign nationality, low level of education, unemployment, nonhome ownership, single-parent family, and overcrowding. Low educational level, unemployment, and lack of home ownership were indirectly associated with the higher mean values of NO_2_ at the statistically significant level (*p* < 0.05). Major resident population and rental housing percentage determined higher levels of PM_10_. Northern regions showed similar results compared to the national level, with the exception of foreign residency that showed direct correlation with the increase of PM_10_. The central regions showed a direct relationship between NO_2_ and PM_10_ levels and higher educational levels and between NO_2_ levels and percentage of single-parent family. In the southern areas, higher NO_2_ levels were correlated with a higher rental housing percentage, as well as higher PM_10_ levels with a higher percentage of unemployment and lower housing density. The study shows high heterogeneity in the findings but confirms the relationship between high educational level and employment with the increased concentration of pollutants. The higher rental housing percentage may increase the pollutants' levels too. The housing density does not seem to be in relationship with NO_2_ and PM_10_ at the national level. The analysis stratified by geographical areas showed that the direction of the correlations was different over time as the analysis was at a national level. The study represents an example of how data from national information systems can provide a preliminary evaluation and be a comparative tool for policy-makers to assess environmental risk factors and social inequalities.

## 1. Introduction

Air pollution, both indoors and outdoors, is caused by contamination by chemical, physical, or biological agents that modify the natural characteristics of the atmosphere.

Primary pollutants are released into the atmosphere from a specific source (such as powders); secondary pollutants are the result of a modification (such as ozone). In particular, primary pollutants derive from human activities or from natural sources.

Primary and secondary pollutants of major public health interest are particulate matter (PM), carbon monoxide (CO), ozone (O_3_), nitrogen dioxide (NO_2_), and sulfur (SO_2_) [1]. The present study focused on two of these substances, i.e., PM and NO_2_.

The PM, due to its physiological and toxicological characteristics, is considered one of the most important pollutants. It is a suspension in the air of solids and liquids with a variable size distribution based on the emission sources. The size of the particles (10 or 2.5 microns of diameter, PM_10_ and PM_2.5_) reflects the toxicological properties, the depth of penetration and deposition in the airways. The chemical composition is an issue of particular health importance; in fact in the PM, polyaromatic hydrocarbons and metals do exist, whose carcinogenicity is certain or probable.

NO_2_ is a toxic gas, irritant for mucous membranes. It is responsible for respiratory diseases (bronchitis, allergies, and irritations). NO_2_ derives both from natural sources (bacteria, volcanoes, and lightning) and from anthropogenic sources (thermoelectric power stations, domestic heating, and petrol and diesel vehicles).

Despite the general reduction in emissions of air pollutants in recent decades, the concentrations are still high and air-quality problems are persistent particularly in urban areas [2].

Epidemiological studies document the harmful effects on human health in the short and long terms; there is a direct correlation between exposure to these risk factors and pathological processes of different nature and intensities [3–7].

In recent years, evidence has focused on vulnerable populations, such as elderly populations, children, patients with disabilities, and disadvantaged socioeconomic classes [8–18]. Health inequalities are unjust differences that determine lack of fairness in health.

In the United States, a bipartisan coalition of academics, researchers, and political activists, called the Congressional Black Caucus, demonstrated that racial minorities and low-income populations were exposed to a higher environmental risk than the general population [19]. Further studies have documented that poorest population tends to concentrate in areas with higher levels of pollution [10, 19–23].

Other surveys have confirmed that the most polluting companies and waste management plants are often located in more deprived residential areas, where green areas are often lacking or not fully accessible [24–28].

Although the literature suggests that increased exposure to pollutants is associated with the ethnicity and socioeconomic status of individuals, many of these studies are limited to population living near monitoring networks. Furthermore, associations are often complex and difficult to refute.

In order to specifically target welfare policies and apply the most appropriate measures to mitigate health inequalities, geographical models are particularly interesting; they explain how these determinants are distributed throughout the country and their temporal trends. These aspects are not yet adequately assessed in Italy. Therefore, the purpose of the present study is to carry out a preliminary investigation on the possible relations between PM_10_ and NO_2_ atmospheric pollution and some of the main characteristics of socioeconomic status (SES) of the population in a subset of Italian municipalities.

## 2. Materials and Methods

### 2.1. Data Collection

The observational study used the monitoring stations as statistical units; the stations were considered at a national level and for single municipalities.

Data from each control unit and relative municipality have been collected:

- Air pollution
- Socioeconomic characteristics
- Demographic characteristics

All the control units that had at least one year of data from 2002 to 2012 were collected for all macro-areas (Table 1).

A database was created in the Excel format, containing all the parameters of the macro-areas available at the local level. Data were imported into the statistical software SPSS 21.0 for analysis.

### 2.2. Data on the Macro-Area: “Atmospheric Pollution”

PM_10_ and NO_2_ concentrations have been considered as indicators of air quality, detected in the network of monitoring stations located throughout the national territory, and contained in the online database BRACE, an Information System of the Italian Institute for Environmental Protection and Research (Istituto Superiore per la Protezione e la Ricerca Ambientale (ISPRA)) [28].

The following statistical parameters available per year and per monitoring station in *μ*g/m^3^ have been extrapolated from raw data available at http://www.brace.sinanet.apat.it/web/struttura.html?p_livello_1=3&p_main=web/sh_dg.inizio&p_scroll=yes:

- Total average annual concentration
- Annual concentration per station: for NO_2_, the data are hourly, equal to 23 per day, about *N*=8298 per year; for PM_10_, the data are daily, one per die, approximately *N*=365 per year

The BRACE statistics report that the average parameter is calculated when the annual series presents at least 50% of the values uniformly distributed over the year [29].

Municipalities that have several monitoring stations in their territory have been considered several times (Table 1).

### 2.3. Data on the Macro-Area: “Socioeconomic Characteristics”

The socioeconomic characteristics (SE) were used for the construction of the Synthetic Deprivation Index (IDS), as provided by Caranci et al. [30].

The IDS was calculated as the sum of the standardized indicators listed in the following:

1. Low educational level: percentage of the population with educational level equal to or less than the elementary license (6 years)
2. Unemployment: percentage of unemployed or job-seeking active population
3. Rented housing: percentage of rented dwellings as a proxy for nonpossession of the dwelling
4. Single-parent family: percentage of single-parent families with cohabiting dependent children
5. Housing density: number of occupants per 100 m^2^ in homes

These variables were collected in the census of 2001 at a national level and aggregated by municipality.

### 2.4. Data on the Macro-Area: “Demographic Characteristics”

Population data were obtained from the online database “Demo” of the National Institute of Statistics (ISTAT) [31]. In particular, demographic data were available at http://demo.istat.it/ and extracted stratified for nationality: Italians and foreign citizens.

The data collected at the municipality level refer to the following:

- Italian resident population on December 31 of each year. The resident population consists of Italian citizens, with residence in the national territory, even if temporarily absent.
- Foreign citizens on December 31 of each year: non-Italian citizens having their usual residence in Italy.

### 2.5. Statistical Analysis

In order to assess the relationship between territorial distribution of NO_2_ and PM_10_ concentrations and the social/material deprivation of the local resident population, Pearson's correlations (*r*) were performed.

The relationship between the NO_2_ and PM_10_ atmospheric pollutants and social/material deprivation for the years 2002 to 2012 was studied by applying linear regression models. The concentration of pollutants was the dependent variable. The following variables were considered independent:

- IDS that includes the five indicators of social and material deprivation
- Number of resident population (Italian citizens)
- Number of foreign citizens
- Geographical area of the municipality (northern, central, and southern regions)

Therefore, twelve regression models (one for each year) were realized with the stepwise backward elimination method with the level of significance at *p* < 0.1.

The goodness of fit of the model was determined by evaluating the *R* ^2^ coefficient. The multicollinearity was checked using variance inflation factors (VIFs). The homogeneity of variance was checked using the scatterplot of the residuals.

In addition, the IDS or the five components of deprivation were considered in the models separately in order to avoid problems of collinearity. The model that showed the highest *R* ^2^ value was chosen and reported in the present article.

Finally, a comparison between the models at national and local levels (northern, central, or southern regions) was made (Table 1). For these groups, defined as macro-regions, the same criteria of significance, analysis, and interpretation were adopted in the national model.

The results of the statistical analyses have been summarized in tables. The level of significance was set at *p* < 0.05.

## 3. Results

The number of monitoring stations of the study was 898, which includes 483 municipalities. Table 1 shows the regional frequency distribution.

The correlation coefficients between IDS and its components towards the annual average values of pollutants per year are shown in Table 2.

The mean annual values of NO_2_ are inversely correlated with the percentage of low education (in all the years considered) and unemployment rate (in 10 out of 11 years). On the contrary, they are directly correlated with the percentage of rented housing and the percentage of single-parent families (9 out of 11 years).

The annual average values of PM_10_ have similar correlations with those of NO_2_: low level of education (in 9 out of 11 years), rented accommodation (in 9 out of 11 years), and unemployment (in 6 out of 11 years), although there is greater discontinuity in terms of significance. Furthermore, the population density per 100 m^2^ is inversely associated with PM_10_ (in 5 out of 11 years).

Linear regression analysis of IDS showed *R* ^2^ values lower than those of the five individual components.  Table 3 shows the results divided by year and by outcome of interest.

The percentage of individuals with a low level of education (covariate referred to as “low education %”) have negative coefficients in all models for the NO_2_ outcomes and are statistically significant since 2006. Instead, for the PM_10_, the association with low educational level changes sign over time and reaches significance in two years: it turns out to be inverse in 2002 (*β* = −0.454, *p*=0.004) and direct from 2010 (*β* = 0.124, *p*=0.020).

The percentage of unemployment was significantly and inversely associated with NO_2_ in 9 out of 11 models. The PM_10_ concentration showed the opposite of the report over time and was statistically significant in four out of 11 models, from 2005 to 2008, with negative values.

The percentage of “rented houses” were directly and significantly associated with both the average values of NO_2_ and PM_10_, respectively, in all and 9 out of 11 years examined.

The covariate monoparenting families had an inverse association with the two pollutants, but this relationship had an inconsistent significance over time.

The independent variable housing density was directly associated, but never significantly, with the annual average value of NO_2_. It was also inversely associated with PM_10_, which was constantly statistically significant from 2009 onwards.

The resident population parameter had positive and statistically significant coefficients (*p* < 0.001) in nine out of eleven models for the dependent variable NO_2_, while for PM_10_, these coefficients were statistically significant in all 11 years but with an inverse association in 4.

The covariate foreign citizens presented an inconstant relation with both pollutants in terms of direction of the association and statistical significance.

The *R* ^2^ of models, related to the NO_2_, ranged from 0.268 to 0.352, while for PM_10_, *R* ^2^ of models ranged from 0.110 to 0.233.

### 3.1. Comparison by Geographical Area

#### 3.1.1. Northern Regions

Table 4 shows the results about northern regions of Italy. Concerning the mean value of NO_2_, statistically significant relationships were observed:

- Direct association with “rented housing” and “resident population”
- Indirect association with the “low level of education” from 2007

The average concentration of PM_10_ results had statistically significant relationships too:

- Direct association with “rented housing” in 7 out of 11 years and “percentage of foreign citizens” in 9 out of 11 years
- Indirect association with the “housing density per 100 m^2^” from 2007 onwards

#### 3.1.2. Central Regions

Table 5 shows results about central regions of Italy. As regards the average value of NO_2_, a direct relationship with the “monoparent families” was observed, statistically significant in 6 out of 11 models, while an inverse association with the low level of education was observed in 5 out of 11 models. The mean PM_10_ concentration showed statistically significant inverse association with a low level of education from 2009 onwards.

#### 3.1.3. Southern Regions

Table 6 shows the models related to Southern Italy. The average value of NO_2_ was directly associated with the rented housing indicators (in 7 out of 11 years) and foreign nationality (in 9 out of 11 years). The average concentration of PM_10_ resulted to be inversely associated with the “housing density” in 5 out of 11 years and directly associated with the percentage of “unemployment.”

## 4. Discussion

The study shows linear correlations between the NO_2_ and PM_10_ concentrations and the indicators of social and material deprivation of the local resident population. The results are in line with the results of previous studies, including some conducted in the United States, North Carolina, and Spain [10, 20, 32]. The study confirms that higher education and employment are related to higher average annual values of PM_10_ and NO_2_, encouraging the interpretation about the association between pollution and anthropogenic activities. This report is also shown in the analysis carried out in the northern regions.

In addition, the areas with a higher number of rented houses are linked to higher average values of PM_10_ and NO_2_. This situation is confirmed in the stratified analysis except for the central region. It could be explained from the fact that younger people often live in rented house and move more for work and for social activities, using motor vehicles, compared to older people. A higher number of residents seem to be associated with an increase of the annual average values of NO_2_ more than of PM_10_, even if this relationship is not confirmed in terms of the direction of relationship and its significance from the statistical point of view. It is therefore necessary to reflect on the fact that a higher number of residents may not correspond to a higher population density. Moreover, always with regard to the number of residents compared to PM_10_, a greater number of residents can lead to an increase in human activities, such as vehicular traffic, which is one of the main sources of the increase of this pollutant. However, this relationship in the stratified analysis by geographical area is not confirmed.

The NO_2_ concentrations at the national level seem to be greater where a greater number of residents do exist, and this is confirmed in the analysis conducted on the regions of Northern Italy; probably, the atmospheric and climatic conditions contribute to a greater consumption of heat in homes or even greater to the presence of industrial combustion plants. A higher number of residents seem to increase the annual average values of NO_2_ more than of PM_10_, even if this relationship is not confirmed both in terms of sign and significance of population density per 100 m^2^. It is therefore necessary to consider that a higher number of residents may not correspond to a high population density. Moreover, a greater number of residents can lead to an increase in human activities, such as vehicular traffic, which is one of the main sources of increase of PM_10_. However, this relationship in the stratified analysis by geographical area is not confirmed. The NO_2_ concentrations at the national level seem to be greater where there are a greater number of residents, and this is confirmed in the analysis conducted on the northern regions; probably, the atmospheric and climatic conditions contribute to a greater consumption of heat in homes or even greater to the presence of industrial combustion plants.

Probably, this variable as well as education, unemployment, rented housing, and single-parent families seem to be indicators influenced by geographical area. In this regard, in some respects, the northern regions seem to reproduce in small what is shown at the national level, while the central and southern regions show situations either reversing or discontinuous over time or changing in the statistical significance level. In conclusion, there is not a common trend, in sign and significance, between national level and the three areas, except for the population density that, at both national and subarea levels, does not show statistically significant relations about average NO_2_.

In the analysis of stratified regression by macro-area, geographical position modifies the effect of some SES components on NO_2_ and PM_10_ levels. The relationships between dependent and independent variables are very different in the three groups in terms of sign, time, and significance and are not imputable to the case. The differences should be analyzed and weighted to reduce social disparities.

The scientific literature reports that the relationships between the various social and environmental risk factors are not always constant and homogeneous over time in the geographical areas and in the territory [10, 20]. Differences documented are spatial and temporal and often depend on the historical social structure, the economic development of individual cities, and their evolution [23].

One of the strength points of our study is the evaluation of relationships between variables not only on a national scale but also for territorial divisions. Moreover, the data refer to the individual local realities and are then aggregated by macro geographical areas.

Another point of strength is that the study included observations from 483 Italian municipalities, around 6% of the total administrative units. Although the sample distribution among regions is not completely homogeneous, it is representative of the Italian municipal situation. In terms of data, northern regions gave the greatest contribution at a national level, considering geographical areas Emilia Romagna and Lombardy in the north, Tuscany in the center, and Puglia and Islands in the south.

Furthermore, the linear regression model was generally suitable to describe the relationship between the dependent (mean NO_2_ and PM_10_ per year) and independent (deprivation index and related indicators) variables.

Considering possible limitations, the study has a cross-sectional design that does not allow to highlight causal links between SES and pollution level. Data collected, thanks to the databases, are not exhaustive to explain the complexity of the causal link between environmental and social inequalities. Moreover, we cannot fully understand whether or not epidemiological measures obtained in the current study represent an under- or overestimation of the true effect and the potential magnitude of sources of bias. So, the results must be considered with care.

Another limiting aspect is the lack of pollution estimates for small areas, which could have led to a misclassification of environmental exposure.

Further variables could be included in the models to explain the relationships. The models show a reduced reliability of relationship using the linear function, in particular for PM_10_ that has an *R* ^2^ that does not exceed the value of 0.233 in the national analysis. In this sense, it is possible that structural equation modeling (SEM) would yield better results than linear regression modeling. Other aspects to consider are the annual quantification of vehicular traffic, the urban setting (presence of parks, industries, etc.), and altitude and proximity to the coast, which could mitigate the effects of dispersed air pollution. Variables such as the nonuse or delay of dental care, often used as a proxy for the economic condition of a family, could be included to describe aspects related to SES.

## 5. Conclusion

The study confirms previous literature about the relationship between SES and atmospheric pollution from PM_10_ and NO_2_. In particular, low level of education, lack of possession of the home, and the population density seem to be related to high levels of PM_10_ and NO_2_. Furthermore, the geographic area appears as a modifier effect that changes the correlation sign between pollutants and SES components. The study represents a proposal for the possible use of data in Italian information systems to discuss environmental and social inequalities.

The models proposed are a starting point for the analysis of the correlation between social determinants of health and environmental risk factors; therefore, we can limit ourselves to general statements about environmental and social inequalities. It is necessary to consider the specific local context in order to find data that are more robust.

## Figures and Tables

**Table 1:** Description of sample size.

Macro-region	Region	No. of municipal districts	No. of air monitoring stations
Northern	Emilia Romagna	57	111
	Friuli Venezia Giulia	24	38
	Liguria	21	56
	Lombardy	86	116
	Piedmont	30	45
	Trentino-South Tyrol	20	30
	Aosta Valley	6	10
	Veneto	43	67
	Total	287	473

Central	Abruzzo	8	13
	Latium	21	42
	Marches	20	31
	Tuscany	36	71
	Umbria	8	21
	Total	93	178

Southern	Basilicata	7	12
	Calabria	7	9
	Campania	6	20
	Molise	6	11
	Puglia	26	61
	Sardinia	26	65
	Sicily	25	69
	Total	103	247

*Total*		**483**	**898**

**Table 2:** Pearson's correlations (*r*) between annual pollutants mean values versus IDS and its five multidimensions per year.

Year	The annual mean of NO_2_							The annual mean of PM_10_						
		Synthetic Deprivation Index	% subjects with low level of education	% unemployment	% nonhome ownership	% single-parent family	Overcrowding 100 m^2^	Synthetic Deprivation Index	% subjects with low level of education	% unemployment	% nonhome ownership	% single-parent family	Overcrowding 100 m^2^
2002	*r*	**0.290**	**−0.364**	**0.192**	**0.463**	**0.263**	**0.290**	**−**0.112	**−0.329**	0.001	**−**0.006	0.150	**−**0.030
	*p*	**0.001**	**<0.001**	**0.034**	**<0.001**	**0.003**	**0.001**	0.435	**0.018**	0.992	0.966	0.294	0.833
2003	*r*	0.106	**−0.435**	**−**0.103	**0.462**	0.093	0.106	**−**0.005	**−**0.001	**−**0.048	0.037	**−**0.138	**−**0.007
	*p*	0.100	**<0.001**	0.108	**<0.001**	0.149	0.100	0.967	0.989	0.663	0.733	0.207	0.948
2004	*r*	0.046	**−0.425**	**−0.196**	**0.480**	0.057	0.046	0.048	**−**0.056	**−**0.055	**0.226**	0.045	**−**0.064
	*p*	0.446	**<0.001**	**0.001**	**<0.001**	0.348	0.446	0.606	0.551	0.559	**0.014**	0.633	0.495
2005	*r*	0.017	**−0.367**	**−0.157**	**0.437**	**0.177**	0.017	**−0.147**	**−0.252**	**−0.316**	**0.325**	0.039	**−0.159**
	*p*	0.755	**<0.001**	**0.004**	**<0.001**	**0.001**	0.755	**0.049**	**0.001**	**<0.001**	**<0.001**	0.607	**0.034**
2006	*r*	**−**0.025	**−0.359**	**−0.201**	**0.373**	**0.151**	**−**0.025	**0.191**	**−0.206**	**−0.337**	**0.236**	**−**0.029	**−0.201**
	*p*	0.605	**<0.001**	**<0.001**	**<0.001**	**0.002**	0.605	**0.001**	**0.001**	**<0.001**	**<0.001**	0.631	**0.001**
2007	*r*	**−**0.039	**−0.356**	**−0.211**	**0.376**	**0.168**	**−**0.039	**−0.130**	**−0.125**	**−0.244**	**0.205**	**−**0.035	**−0.198**
	*p*	0.347	**<0.001**	**<0.001**	**<0.001**	**<0.001**	0.347	**0.011**	**0.014**	**<0.001**	**<0.001**	0.490	**<0.001**
2008	*r*	0.056	**−0.340**	**−0.118**	**0.413**	**0.188**	0.056	0.050	**−0.116**	**−0.115**	**0.293**	0.042	**−**0.029
	*p*	0.179	**<0.001**	**0.004**	**<0.001**	**<0.001**	0.179	0.310	**0.018**	**0.020**	**<0.001**	0.396	0.551
2009	*r*	0.055	**−0.388**	**−0.115**	**0.429**	**0.220**	0.055	0.017	**−0.164**	**−**0.091	**0.289**	**0.112**	**−**0.082
	*p*	0.182	**<0.001**	**0.005**	**<0.001**	**<0.001**	0.182	0.714	**<0.001**	0.050	**<0.001**	**0.016**	0.080
2010	*r*	0.060	**−0.396**	**−0.234**	**0.387**	**0.165**	0.060	0.059	**−0.110**	**−**0.049	**0.287**	**0.109**	**−**0.077
	*p*	0.140	**<0.001**	**<0.001**	**<0.001**	**<0.001**	0.140	0.199	**0.017**	0.283	**<0.001**	**0.017**	0.093
2011	*r*	**−**0.065	**−0.456**	**−0.259**	**0.465**	**0.196**	**−**0.065	**−0.163**	**−0.189**	**−0.251**	**0.262**	0.008	**−0.269**
	*p*	0.129	**<0.001**	**<0.001**	**<0.001**	**<0.001**	0.129	**<0.001**	**<0.001**	**<0.001**	**<0.001**	0.862	**<0.001**
2012	*r*	**−**0.027	**−0.475**	**−0.219**	**0.477**	**0.221**	**−**0.027	**−0.184**	**−0.220**	**−0.283**	**0.277**	0.013	**−0.273**
	*p*	0.529	**<0.001**	**<0.001**	**<0.001**	**<0.001**	0.529	**<0.001**	**<0.001**	**<0.001**	**<0.001**	0.770	**<0.001**

Values whose *p* < 0.05 are given in bold.

**Table 3:** Linear regression models of dependent variables “annual mean of NO_2_” and “annual mean of PM_10_.”

Pollutants	Model for year	Covariates														
		% subjects with low level of education		% unemployment		% nonhome ownership		% single-parent family		Overcrowding 100 m^2^		Resident population		Foreign citizen		*R* ^2^ adj.
		*β*	*p*	*β*	*p*	*β*	*p*	*β*	*p*	*β*	*p*	*β*	*p*	*β*	*p*	
The annual mean of NO_2_	2002	**−**0.089	0.332	0.061	0.625	**0.324**	**<0.001**	**−**0.091	0.328	0.144	0.132	**0.333**	**<0.001**	0.043	0.882	0.294
	2003	**−**0.126	0.079	**−**0.118	0.056	**0.338**	**<0.001**	**−0.168**	**0.006**	**−**0.239	0.811	**0.336**	**<0.001**	**−**0.272	0.138	0.340
	2004	**−**0.113	0.087	**−0.141**	**0.007**	**0.303**	**<0.001**	**−0.150**	**0.005**	0.004	0.946	**0.338**	**<0.001**	**−**0.128	0.432	0.346
	2005	**−**0.075	0.265	**−0.149**	**0.002**	**0.324**	**<0.001**	**−**0.044	0.402	0.013	0.835	**0.296**	**<0.001**	**−**0.141	0.343	0.280
	2006	**−0.12**	**0.036**	**−0.198**	**<0.001**	**0.256**	**<0.001**	**−**0.101	0.056	0.035	0.551	**0.310**	**<0.001**	**−**0.073	0.641	0.277
	2007	**−0.128**	**0.008**	**−0.231**	**<0.001**	**0.254**	**<0.001**	**−0.088**	**0.047**	0.004	0.937	**0.296**	**<0.001**	**−**0.206	0.125	0.281
	2008	**−0.120**	**0.014**	**−0.223**	**<0.001**	**0.306**	**<0.001**	**−0.096**	**0.036**	0.031	0.543	**0.619**	**<0.001**	**−0.366**	**0.012**	0.268
	2009	**−0.118**	**0.007**	**−0.152**	**<0.001**	**0.280**	**<0.001**	**−**0.061	0.157	0.033	0.488	**0.269**	**<0.001**	**−**0.219	0.139	0.285
	2010	**−0.173**	**<0.001**	**−0.199**	**<0.001**	**0.249**	**<0.001**	**−**0.076	0.068	0.054	0.224	0.053	0.744	**0.254**	**<0.001**	0.291
	2011	**−0.204**	**<0.001**	**−0.182**	**<0.001**	**0.266**	**<0.001**	**−**0.074	0.073	0.040	0.350	**−**0.066	0.702	**0.260**	**<0.001**	0.352
	2012	**−0.231**	**<0.001**	**−0.179**	**<0.001**	**0.300**	**<0.001**	**−0.085**	**0.049**	0.015	0.732	**0.223**	**<0.001**	0.002	0.989	0.349

The annual mean of PM_10_	2002	**−0.454**	**0.004**	0.457	0.094	**−**0.146	0.460	**−**0.055	0.793	**−**0.442	0.099	**0.240**	**<0.001**	**−**0.106	0.924	0.110
	2003	0.074	0.597	**−**0.066	0.544	0.072	0.661	**−0.321**	**0.006**	**−**0.086	0.667	**0.402**	**<0.001**	**−**0.441	0.324	0.126
	2004	0.189	0.058	0.198	0.115	**0.267**	**0.009**	0.017	0.875	**−0.211**	**0.020**	**−0.413**	**<0.001**	0.401	**<0.001**	0.185
	2005	0.007	0.938	**−0.294**	**<0.001**	**0.303**	**<0.001**	**−**0.011	0.884	**−**0.057	0.517	**−0.315**	**<0.001**	0.111	0.122	0.182
	2006	**−**0.023	0.765	**−0.211**	**0.004**	**0.253**	**<0.001**	**−0.144**	**0.021**	**−**0.123	0.086	**0.011**	**<0.001**	**0.197**	**0.001**	0.193
	2007	**−**0.033	0.616	**−0.127**	**0.035**	**0.261**	**<0.001**	**−0.188**	**0.001**	**−0.200**	**0.001**	**0.197**	**<0.001**	**−**0.096	0.641	0.151
	2008	0.064	0.294	**−0.168**	**<0.001**	**0.331**	**<0.001**	**−0.132**	**0.017**	**−**0.069	0.286	**0.157**	**<0.001**	**−**0.114	0.572	0.134
	2009	0.012	0.848	**−**0.023	0.680	**0.292**	**<0.001**	**−**0.075	0.143	**−0.185**	**<0.001**	**0.128**	**<0.001**	**−**0.088	0.643	0.118
	2010	**0.124**	**0.020**	0.014	0.817	**0.375**	**<0.001**	**−**0.046	0.441	**−0.211**	**<0.001**	**0.100**	**<0.001**	**−**0.113	0.538	0.117
	2011	**−**0.046	0.430	**−**0.008	0.875	**0.350**	**<0.001**	**−0.187**	**<0.001**	**−0.342**	**<0.001**	**−0.308**	**<0.001**	**0.160**	**<0.001**	0.205
	2012	**−**0.094	0.094	**−**0.012	0.836	**0.330**	**<0.001**	**−0.202**	**<0.001**	**−0.310**	**<0.001**	**−0.449**	**0.032**	**0.584**	**0.005**	0.233

Values whose *p* < 0.05 are given in bold.

**Table 4:** Linear regression models of dependent variables “annual mean of NO_2_” and “annual mean of PM_10_” in Northern Italian regions.

Pollutants	Model for year	Covariates														
		% subjects with low level of education		% unemployment		% nonhome ownership		% single-parent family		Overcrowding 100 m^2^		Resident population		Foreign citizen		*R* ^2^ adj.
		*β*	*p*	*β*	*p*	*β*	*p*	*β*	*p*	*β*	*p*	*β*	*p*	*β*	*p*	
The annual mean of NO_2_	2002	**−**0.081	0.514	0.064	0.604	0.118	0.328	**−**0.069	0.537	0.108	0.290	**0.580**	**<0.001**	**−**0.554	0.105	0.327
	2003	**−**0.124	0.154	**−**0.138	0.079	**0.287**	**<0.001**	**−0.153**	**0.035**	**−**0.012	0.864	**1.101**	**<0.001**	**−0.720**	**0.012**	0.300
	2004	**−**0.117	0.124	**−0.175**	**0.008**	**0.283**	**<0.001**	**−0.129**	**0.033**	0.001	0.996	**1.058**	**<0.001**	**−0.661**	**0.005**	0.342
	2005	**−**0.102	0.243	**−0.181**	**0.019**	**0.302**	**<0.001**	**−0.153**	**0.033**	0.094	0.172	**0.905**	**0.001**	**−0.594**	**0.023**	0.235
	2006	**−**0.111	0.154	**−**0.124	0.064	**0.179**	**0.008**	**−**0.095	0.150	0.063	0.306	**0.830**	**0.001**	**−**0.460	0.055	0.215
	2007	**−0.134**	**0.038**	**−0.130**	**0.020**	**0.145**	**0.022**	**−**0.059	0.288	0.013	0.801	**0.936**	**<0.001**	**−0.580**	**0.004**	0.262
	2008	**−0.200**	**0.001**	**−**0.104	0.052	**0.152**	**0.011**	**−**0.087	0.101	0.025	0.603	**0.989**	**<0.001**	**−0.635**	**0.002**	0.318
	2009	**−0.215**	**<0.001**	**−0.138**	**0.008**	**0.231**	**<0.001**	**−**0.089	0.087	0.058	0.227	**0.837**	**<0.001**	**−0.454**	**0.024**	0.376
	2010	**−0.197**	**0.001**	**−0.109**	**0.032**	**0.281**	**<0.001**	**−**0.094	0.059	0.049	0.303	**0.710**	**0.001**	**−**0.359	0.088	0.380
	2011	**−0.125**	**0.048**	**−**0.085	0.097	**0.369**	**<0.001**	**−0.113**	**0.027**	0.095	0.050	**0.822**	**<0.001**	**−0.484**	**0.022**	0.424
	2012	**−0.186**	**0.004**	**−**0.064	0.246	**0.256**	**<0.001**	**−**0.068	0.219	0.060	0.240	**0.837**	**<0.001**	**−0.516**	**0.015**	0.390

The annual mean of PM_10_	2002	**−**0.206	0.287	**−**0.240	0.253	**−0.461**	**0.013**	**−**0.285	0.165	**−**0.156	0.573	**−**0.162	0.913	**0.707**	**<0.001**	0.353
	2003	**0.421**	**<0.001**	**−**0.082	0.515	**−**0.088	0.587	**−**0.054	0.733	**−**0.161	0.163	**0.603**	**<0.001**	**−**0.924	0.273	0.454
	2004	**0.350**	**0.002**	**−**0.072	0.477	**0.242**	**0.032**	0.002	0.987	**−**0.164	0.122	**0.560**	**<0.001**	**−**0.080	0.851	0.357
	2005	**−**0.008	0.947	**−**0.138	0.146	**0.216**	**0.027**	**−**0.146	0.153	0.070	0.454	**−**0.025	0.948	**0.201**	**0.040**	0.100
	2006	0.003	0.973	**−**0.034	0.673	**0.235**	**0.013**	**−0.296**	**0.001**	0.064	0.439	**−**0.078	0.805	**0.216**	**0.013**	0.154
	2007	**−**0.057	0.531	**−**0.036	0.665	0.115	0.170	**−0.239**	**0.001**	**−0.158**	**0.029**	**−**0.363	0.139	**0.249**	**0.001**	0.131
	2008	0.024	0.774	**−**0.014	0.860	0.115	0.152	**−**0.091	0.176	**−0.159**	**0.018**	**−0.883**	**<0.001**	**1.132**	**<0.001**	0.146
	2009	0.069	0.416	**−**0.034	0.640	**0.257**	**0.001**	**−0.157**	**0.035**	**−0.119**	**0.069**	**−0.682**	**0.005**	**0.863**	**<0.001**	0.166
	2010	0.080	0.317	0.036	0.605	**0.298**	**<0.001**	**−**0.130	0.065	**−0.189**	**0.003**	**−0.765**	**0.002**	**0.904**	**<0.001**	0.190
	2011	0.018	0.831	**−**0.025	0.708	**0.357**	**<0.001**	**−0.254**	**<0.001**	**−0.187**	**0.002**	**−**0.384	0.172	**0.220**	**0.001**	0.223
	2012	**−**0.023	0.778	**−**0.074	0.286	**0.396**	**<0.001**	**−0.237**	**0.001**	**−0.161**	**0.011**	**−**0.491	0.088	**0.667**	**0.020**	0.216

Values whose *p* < 0.05 are given in bold.

**Table 5:** Linear regression models of dependent variables “annual mean of NO_2_” and “annual mean of PM_10_” in Central Italian regions.

Pollutants	Model for year	Covariates														
		% subjects with low level of education		% unemployment		% nonhome ownership		% single-parent family		Overcrowding 100 m^2^		Resident population		Foreign citizen		*R* ^2^ adj.
		*β*	*p*	*β*	*p*	*β*	*p*	*β*	*p*	*β*	*p*	*β*	*p*	*β*	*p*	
The annual mean of NO_2_	2002	**−0.561**	**0.001**	**−**0.114	0.514	**−**0.301	0.568	0.283	0.527	0.244	0.172	2.626	0.557	**−**0.117	0.706	0.293
	2003	**−0.515**	**<0.001**	0.092	0.665	**−**0.394	0.224	0.270	0.444	**−**0.060	0.682	0.250	0.182	**−**0.203	0.933	0.248
	2004	**−**0.092	0.758	0.133	0.306	**−**0.237	0.556	**0.496**	**<0.001**	**−**0.223	0.227	**−**0.421	0.886	0.148	0.436	0.230
	2005	**−0.146**	**<0.001**	**−**0.069	0.647	0.003	0.993	0.126	0.680	**−**0.043	0.804	0.263	0.133	**−**2.194	0.313	0.197
	2006	**−0.310**	**0.046**	**−**0.065	0.668	**−**0.113	0.659	0.130	0.637	0.008	0.965	0.267	0.085	**−**0.723	0.791	0.260
	2007	**−**0.097	0.511	0.044	0.696	0.014	0.928	**0.305**	**0.004**	0.123	0.189	**−**1.420	0.347	**0.225**	**0.034**	0.221
	2008	**−**0.070	0.671	0.009	0.941	0.101	0.427	**0.402**	**<0.001**	0.098	0.308	**−**1.903	0.290	0.136	0.206	0.155
	2009	**−**0.182	0.182	0.092	0.321	0.036	0.805	**0.474**	**<0.001**	**−**0.006	0.956	**−**2.684	0.123	0.147	0.155	0.219
	2010	0.071	0.688	0.046	0.686	0.087	0.492	**0.419**	**<0.001**	0.108	0.238	**−**2.494	0.176	0.158	0.130	0.169
	2011	**−0.374**	**<0.001**	0.161	0.076	0.032	0.819	0.203	0.181	**−**0.014	0.898	**−4.119**	**0.010**	**4.309**	**0.007**	0.333
	2012	**−**0.102	0.564	**0.187**	**0.031**	0.200	0.105	**0.451**	**<0.001**	**−**0.024	0.816	**−4.580**	**0.010**	**4.673**	**0.008**	0.346

The annual mean of PM_10_	2002	**−**0.591	0.138	**0.681**	**0.021**	0.001	0.999	**−**1.118	0.418	0.001	0.999	0.001	0.999	**−**0.247	0.374	0.404
	2003	**−**1.046	0.073	**−**0.099	0.806	**−**0.897	0.119	0.049	0.952	0.314	0.272	1.120	0.884	**−**0.703	0.164	0.100
	2004	**−**0.438	0.053	0.133	0.852	**−**0.607	0.302	**−**0.308	0.591	0.152	0.554	**−**0.399	0.335	2.456	0.582	0.147
	2005	**−**0.402	0.079	**−**0.121	0.611	**−**0.065	0.928	0.082	0.911	0.570	0.128	1.188	0.902	**−**0.252	0.386	0.115
	2006	**−**0.104	0.785	0.129	0.385	0.347	0.079	**−**0.179	0.711	**0.891**	**<0.001**	**−0.695**	**0.008**	3.895	0.443	0.406
	2007	**−**0.096	0.623	**−**0.088	0.583	0.031	0.864	0.165	0.126	**0.261**	**0.012**	1.732	0.428	**−**0.140	0.344	0.058
	2008	**−**0.093	0.489	0.013	0.950	0.123	0.409	**0.263**	**0.006**	0.106	0.289	0.378	0.880	**−**0.133	0.322	0.060
	2009	**−0.391**	**<0.001**	**−**0.173	0.092	0.138	0.391	0.218	0.217	0.136	0.249	0.327	0.889	**−**0.145	0.216	0.103
	2010	**−0.359**	**0.002**	**−**0.161	0.109	0.109	0.512	0.174	0.329	0.158	0.185	**−**0.194	0.089	**−**0.068	0.979	0.065
	2011	**−0.280**	**0.003**	0.082	0.407	**−**0.086	0.602	**−**0.022	0.912	**−**0.049	0.693	**−**0.110	0.343	1.849	0.321	0.070
	2012	**−0.357**	**<0.001**	0.120	0.245	**−**0.112	0.497	0.096	0.614	**−**0.035	0.776	**−**0.148	0.187	1.782	0.322	0.120

Values whose *p* < 0.05 are given in bold.

**Table 6:** Linear regression models of dependent variables “annual mean of NO_2_” and “annual mean of PM_10_” in Southern Italian regions.

Pollutants	Model for year	Covariates														
		% subjects with low level of education		% unemployment		% nonhome ownership		% single-parent family		Overcrowding 100 m^2^		Resident population		Foreign citizen		*R* ^2^ adj.
		*β*	*p*	*β*	*p*	*β*	*p*	*β*	*p*	*β*	*p*	*β*	*p*	*β*	*p*	
The annual mean of NO_2_	2002	0.144	0.637	0.068	0.983	**0.642**	**0.004**	**−**0.259	0.204	**−**0.580	0.104	0.851	0.597	**−**0.381	0.189	0.760
	2003	0.093	0.563	**−0.626**	**0.002**	**0.691**	**<0.001**	**−**0.169	0.153	**−**0.086	0.806	0.085	0.916	**0.718**	**0.002**	0.723
	2004	0.093	0.562	**−**0.127	0.484	**−**0.241	0.551	**0.204**	**0.025**	0.116	0.660	**0.880**	**<0.001**	**−**5.897	0.116	0.765
	2005	**−**0.032	0.777	**0.206**	**0.025**	**0.397**	**<0.001**	**0.138**	**0.080**	**−**0.098	0.469	**−**0.289	0.440	**0.192**	**0.085**	0.472
	2006	**−**0.059	0.443	0.085	0.463	**0.579**	**<0.001**	**−**0.056	0.611	**−**0.067	0.615	**−1.252**	**0.008**	**1.253**	**0.005**	0.319
	2007	**−**0.182	0.076	**−**0.060	0.516	**0.454**	**0.002**	**−0.255**	**0.014**	**−**0.190	0.080	**−**0.397	0.361	**0.364**	**0.001**	0.361
	2008	0.020	0.803	0.062	0.405	**0.283**	**0.004**	0.016	0.855	0.013	0.907	**−**0.369	0.333	**0.439**	**<0.001**	0.458
	2009	**−**0.071	0.400	0.041	0.564	**0.233**	**0.023**	**−**0.011	0.906	0.058	0.540	**−**0.621	0.097	**1.071**	**0.003**	0.421
	2010	**−0.150**	**0.045**	**−**0.016	0.860	0.073	0.535	**−**0.156	0.134	0.045	0.720	**−**0.175	0.668	**0.388**	**<0.001**	0.187
	2011	**−**0.110	0.190	**−**0.019	0.821	0.015	0.911	**−**0.034	0.739	0.116	0.123	0.047	0.796	**0.468**	**<0.001**	0.214
	2012	**−0.203**	**0.018**	0.001	0.996	0.160	0.088	**−0.168**	**0.038**	0.020	0.834	**−**0.195	0.524	**0.447**	**<0.001**	0.346

The annual mean of PM_10_	2002	**−**0.380	0.715	0.001	0.999	0.001	0.999	**−**0.171	0.687	0.001	0.999	0.001	0.999	0.281	0.403	0.001
	2003	**−**0.228	0.258	**0.671**	**0.001**	**−**0.731	0.139	0.089	0.748	0.276	0.574	**−**0.238	0.869	0.944	0.153	0.421
	2004	0.245	0.531	**1.216**	**0.023**	**−1.883**	**0.001**	0.072	0.863	**−**0.220	0.376	**21.085**	**0.002**	**−19.913**	**0.003**	0.463
	2005	0.153	0.408	**−**0.039	0.864	**−0.650**	**0.021**	**0.511**	**0.001**	0.092	0.740	**0.873**	**0.001**	**−**5.069	0.155	0.315
	2006	**−**0.024	0.891	0.016	0.900	0.135	0.432	**−**0.121	0.336	**−0.251**	**0.027**	**0.362**	**0.002**	0.010	0.986	0.078
	2007	**−**0.078	0.571	**0.288**	**0.002**	**0.432**	**<0.001**	**−**0.072	0.495	**−0.277**	**0.008**	0.041	0.940	0.078	0.584	0.223
	2008	**−**0.015	0.895	0.035	0.719	**0.664**	**<0.001**	**−**0.030	0.700	**−**0.080	0.494	0.177	0.163	**−**0.001	0.999	0.436
	2009	**−**0.104	0.321	0.133	0.120	**0.253**	**0.040**	**−**0.023	0.827	**−0.242**	**0.008**	**−**0.269	0.542	**0.429**	**<0.001**	0.286
	2010	**−**0.002	0.983	**0.370**	**<0.001**	0.006	0.967	0.035	0.697	**−0.176**	**0.024**	0.001	0.999	**0.479**	**<0.001**	0.419
	2011	**−**0.063	0.633	**0.194**	**0.018**	0.105	0.422	**−**0.089	0.325	**−0.289**	**<0.001**	**0.366**	**<0.001**	0.142	0.586	0.197
	2012	**−**0.061	0.579	**0.167**	**0.043**	0.212	0.094	**−0.238**	**0.011**	**−0.411**	**<0.001**	0.062	0.855	**0.217**	**0.033**	0.165

Values whose *p* < 0.05 are given in bold.

## Data Availability

The datasets supporting the conclusions of the study are available from the corresponding author upon request.
